# Educational and health outcomes of schoolchildren in local authority care in Scotland: a retrospective record linkage study.

**DOI:** 10.23889/ijpds.v7i3.2020

**Published:** 2022-08-25

**Authors:** Michael Fleming

**Affiliations:** 1 University of Glasgow

## Objectives

Looked-after-children are defined as children who are in the care of their local authority. Previous studies have reported that looked-after-children have poorer mental and physical health, increased behavioural problems, and increased self-harm and mortality compared to peers. They also experience poorer educational outcomes yet population wide research into the latter is lacking, particularly in the UK. Education and health share a bidirectional relationship therefore it is important to dually investigate both outcomes. Our study aimed to compare educational and health outcomes for looked-after-children with peers, adjusting for sociodemographic, maternity and comorbidity confounders.

## Approach

Linkage of nine Scotland-wide databases, covering dispensed prescriptions, hospital admissions, maternity records, death certificates, annual pupil census, examinations, school absences/exclusions, unemployment, and looked-after-children provided retrospective data on 715,111 children attending Scottish schools between 2009 and 2012.

## Results

Compared to peers, 13,898 (1.9%) looked-after-children were more likely to be absent and excluded from school, have special educational need and neurodevelopmental multimorbidity, achieve the lowest level of academic attainment, and be unemployed after leaving school. They were more likely to require treatment for epilepsy, attention deficit hyperactivity disorder and depression, be hospitalised overall, for injury and self-harm, and die prematurely. Compared to children looked after at home, children looked after away from home had less absenteeism, less exclusion, less unemployment, and better attainment. Therefore, amongst those in care, being cared for away from home appeared to be a protective factor resulting in better educational outcomes.

## Conclusion

Looked-after-children had poorer health and educational outcomes than peers independent of increased neurodevelopmental conditions and special educational need. Further work is required to understand whether poorer outcomes relate to reasons for entering care, including maltreatment and adverse childhood events, neurodevelopmental vulnerabilities, or characteristics of the care system.

